# Drug Use and Sex Work: Competing Risk Factors for Newly Acquired HIV in Yunnan, China

**DOI:** 10.1371/journal.pone.0059050

**Published:** 2013-03-28

**Authors:** Junjie Xu, M. Kumi Smith, Guowei Ding, Jennifer Chu, Haibo Wang, Qinghua Li, Dongfang Chang, Guixiang Wang, Hong Shang, Yan Jiang, Ning Wang

**Affiliations:** 1 Key Laboratory of AIDS Immunology of Ministry of Health, Department of Laboratory Medicine, No.1 Hospital of China Medical University, Shenyang, China; 2 National Center for AIDS/STD Control and Prevention, Chinese Center for Disease Control and Prevention, Beijing, China; 3 Department of Epidemiology, Gillings School of Global Public Health, University of North Carolina, Chapel Hill, North Carolina, United States of America; 4 Honghe Prefecture Center for Disease Control and Prevention, Mengzi City, Yunnan, China; 5 Kaiyuan City Center for Disease Control and Prevention, Kaiyuan, Yunnan, China; 6 National AIDS Reference Laboratory, National Center for AIDS/STD Control and Prevention, Beijing, China; Indiana University and Moi University, United States of America

## Abstract

**Objective:**

To investigate the HIV incidence and its related factors among female sex workers (FSWs) in a high prevalence area where injection drug use is also widely documented.

**Method:**

A cross-sectional study of 1642 female sex workers (FSWs) was conducted in Honghe Prefecture of Yunnan Province. Interviewed-questionnaires were administrated to collect information on sexual partnerships, condom use and illicit drug using behaviors etc. Blood samples were collected to test for HIV antibodies, and all HIV seropositive specimens were tested with the BED IgG capture-based enzyme immunosorbent assay (BED-CEIA) to distinguish between new and established HIV infection (<153 days).

**Results:**

15.9% (261/1642) of participants reported ever having used drugs, and 7.4% had injected in recent 3 months. The overall HIV prevalence was 10.2% (168/1642), among which 16.7% (28/168) were identified as recent infections using BED-CEIA. The crude HIV incidence estimated from BED-CEIA results was 4.4 (95%CI 2.8–6.0) /100 person years (PY). Multivariate logistic analysis showed that an illicit drug using history (by either self-reporting or urine opiates testing) was both significant risk factors both for HIV established and recent infection (each p<0.05). Drug using FSWs (DU-FSW) reported more male clients in the previous week, and had significantly higher prevalence of HIV, chlamydia trachomatis and HSV-2 as compared to non DU-FSW (each p<0.05).

**Conclusion:**

Our results show that a history of drug use poses significant risks for both new and established HIV infection among FSWs, and that HIV-incidence among Honghe FSWs is relatively high compared to similar populations. Comprehensive interventions targeted at DU-FSWs' injection drug using and high risk sexual behaviors are urgently needed to reduce the rapid spread of HIV epidemic.

## Introduction

Worldwide estimated number of people living with HIV or AIDS (PLWHs) is about 34 million, among which unprotected heterosexual intercourse is the predominant mode of HIV transmission [1], [2]. Female sex workers (FSWs) and their male clients have been identified as core populations for the rapid spread of HIV epidemic in various epidemic settings including East Asia [3], [4] and some African countries [5], [6].

The earliest HIV cases identified in China were among intravenous injection drug users, and many such cases were geographically concentrated in Yunnan province due to its proximity to the opium producing regions of Burma, Vietnam, and Laos (collectively known as the Golden Triangle). Throughout the 1990's and early 2000's injection drug remained the dominant mode of HIV transmission in China [7] however in 2007 heterosexual contact replaced injection drug use as the dominant route of HIV transmission. By 2011 heterosexual transmission represented over half (52.2%) of all new infections that year [8]. The rise of risky sex in the context of the commercial sex industry has therefore become the focus of HIV control efforts [9], and the rapid growth of the commercial sex industry – 160-fold since 1985 – demands interventions that can simultaneously scale up while preferentially targeting the highest risk groups first. While overall HIV prevalence among FSWs has remained relatively low at a median of 0.6% [10], prevalence rates as high as 10.3–13.0% [11], [12], [13] have been reported in “hot spot” areas.

Prevalent injection drug use and sex work have both contributed to the HIV epidemic in Yunnan, where 22% of new HIV/AIDS cases in the country are identified annually despite only making up 3.5% of the national population [8], [14]. HIV surveillance data from Yunnan Centers for Disease Control and Prevention (CDC) show that the male to female ratio among reported HIV-infected cases decreased from 13∶1 to 1.9∶1 between 1996–2006, suggesting that transmission is rapidly shifting from the predominantly male drug using populations to the heterosexual population[15]. Though part of this change may reflect changes in reporting and surveillance practices over time, the dramatic change in a relatively short time suggests that HIV is affecting new populations with diverse risk profiles.

The link between illicit drug use behavior and commercial sex has been well investigated [16], [17], [18], [19]. Our previous studies among FSWs in Yunnan have shown that up to 14.1% of them reported ever having used drugs [20], [21], and over a quarter of male injection drug users interviewed in the same area reported sex with FSWs within the last 6 months [22]. The salience of drug use as a risk factor for HIV infection among FSWs is well established [20], [21], [23], [24]. However, in this study we seek to understand the drivers of recent versus established HIV infection in FSW with the use of BED-capture-based enzyme immunosorbent assay (BED-CEIA) [25], [26], [27], [28] in order to identify possible trends in changing modes of HIV acquisition. This type of analysis also allows us to explore the potential role that illicit drug use plays in the relationship between commercial sexual behaviors and HIV infection in such a way to better inform interventions targeting the dual risk behaviors of drug-using FSW.

The analysis uses data from a large scale cross-sectional study conducted between March 2006 and April 2007 in the cities of Gejiu and Kaiyuan of Honghe Prefecture in southern Yunnan province. Both cities are situated on major heroin trafficking routes into China. HIV prevalence among surveyed IDU in Honghe ranged from 59.9–60.4% between 2003 and 2007 [22], [29], and from 8.3% to 10.3% among FSWs between 2006 and 2007 [13], [20].

## Methods

### Study Design and Study Population

During 2006–2007, outreach workers for the study recruited local FSWs from various commercial sex venues including karaoke halls, hair and beauty salon, night clubs to participate in this study. Inclusion criteria for FSWs participants included: 1) residing in Gejiu or Kaiyuan at the time of interview, 2) at least 16 years of age, 3) a reported a history of commercial sex in the last three months, and 4) willing and able to provide written informed consent. A Community Advisory Boards (CAB) made up of brothel managers, retired policemen and active FSWs were set up prior to survey work to monitor and inform study procedures such as questionnaire design and sampling methods. Members of the CAB were encouraged to distribute study-related information to FSWs and to refer FSWs in the community to participate in the study.

### Ethics Statement

Eligible FSWs took part in private face to face interview with trained staff from the local CDC's and provided information on demographics, sexual behaviors, HIV/STD knowledge, and drug use history. Trained medical staff collected blood specimens, urine and cervical-vaginal secretion swabs from each participant. All participants provided written informed consent before attending this study, and each of FSWs had the ability to decline or withdraw from this survey. The questionnaires and written consent document were separately kept in locked cupboards at the study sites, and unrelated persons cannot access on them. The study process and content were approved by the ethics committee of the National Center for AIDS/STD Control and Prevention (NIAIDS) of the Chinese Center for Disease Control and Prevention (CDC) and the ethics committee of NIAIDS Prevention Science Review Committee (PSRC).

### Laboratory Methods

Blood specimens were tested for antibodies against HIV, herpes simplex virus (HSV)-2 and syphilis at related labs in Gejiu city CDC, Kaiyuan city CDC and Yunnan provincial CDC. HIV antibody was tested by enzyme linked immunosorbent assay (ELISA; OrganonTeknika, Boxtel,o., Ltd., the Netherlands), and positive tests were confirmed by HIV-1/2 Western blot assay (HIV Blot 2.2 WB; Genelabs Diagnostics, Singapore). Herpes simplex virus (HSV)-2 antibodies were tested with ELISA (Herpes Select-2 ELISA IgG; Focus Technologies, Cypress, CA). Testing for *Treponemapallidum* was conducted with rapid plasma reagin (RPR Diagnosis t; Xinjiang Xindi, China), and samples positive for RPR were confirmed by *T. pallidum particle assay* (TPPA Serodia; Fujirebio, Inc., Fuji, Japan). Subjects with plasma positive results for both TPPA and RPR were considered currently infected with syphilis. Wet mounts were made from vaginal secretion swabs at the study site by trained staff. Samples were classified as *trichomonas vaginalis* positive if motile organisms were seen. Cervical swab specimens were tested for nucleic acid of *Neisseria gonorrhoeae* (*NG.*) and *chlamydia trachomatis* (*CT.*) by polymerase chain reaction (Amplicor). Each urine specimen was tested for opiates by morphine gold-conjugate test strip (Acon MOP; Acon Biotech, Hangzhou, China) method at the study site.

Blood samples tested positive by WB were selected to test for recent infection by BED-CEIA (Calypte Biomedical Corporation, Rockville, MD, USA). Specimens with ODn more than 1.2 were considered established infected (>153 days), and specimens with initial ODn<1.2 were tested in triplicate to confirm their ODn values. If the median ODn value of all three tests was <0.8, the specimen was considered recently infected (153 days), otherwise, the specimen was classified as chronic infection. Blood specimens were well collected and transported from study sites to AIDS reference laboratory, China CDC for BED-CEIA testing. Samples were optimally stored in low temperature laboratory freezer (minus eighty degree temperature) before carrying out BED-CEIA testing.

### Statistical Analysis

Data from questionnaires and laboratory tests results were entered into and managed by a DataFax system (Clinical DataFax Systems, Hamilton, ON, Canada), and analysis was conducted with SAS 9.1 software (SAS Institute Inc., Cary, North Carolina, USA). Crude BED-CEIA estimated HIV incidence was calculated using the formula recommended by the United States CDC: I = 100×[(365/w)×*N^inc^*]/[*N^neg^*+ (365/w)×(*N^inc^*/2)] [30], in which w is the window period (153 days), N^inc^ is the number of new HIV infections as determined by BED-CEIA, and N^neg^ is the total number of HIV seronegative subjects. The 95% confidence intervals (CI) for estimated BED-CEIA incidence were calculated by: 95% CI = I±1.96(I/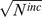) [30]. McDougal-adjusted HIV incidence [26], and Hargrove-adjusted HIV incidence [27] were calculated according to their respective formulas. Univariate logistic analyses were firstly used to explore factors associated with new or established infections. Then, we performed multivariate logistic regression analysis to obtain the adjusted association between different variables and HIV infection status (by adjusting study sites, residence places, and current ages, which have P-values of less than 0.2 when checking the association of those variables with HIV infection status [recent HIV infection or established HIV infection]). Chi-square tests were used to compare categorical values between groups and t-test or Mann-Whitney U tests were used for continuous variables.

## Results

### Sociodemographic Characteristics

One-thousand six hundred and sixty (1660) eligible FSWs were approached to take part in this study, among which 1642(98.9%) took part, with a refusal rate of1.1%. Participants were mainly recruited from karaoke halls (46.4%) and hair salons (23.1%), had a median age of 25 years, and were predominantly of Han ethnicity (64.6%). 682(41.5%) had received less than 6 years of education. 848(51.6%) were never married, while 180(11.0%) were married, 294(17.9%) were divorced, 238(14.5%) were cohabitating with a partner. Over half (57.1%) of the participants were from other prefectures in Yunnan Province, and 276(16.8%) were from other provinces (Table 1).

**Table 1:** Category of recruited Honghe FSWs participants by working locations.

Category of FSWs by working locations	N	Proportion (%)
Karaoke Halls	762	46.4
Hair and beauty salon	379	23.1
Night clubs	145	8.8
Sauna	98	6.0
Rented room	91	5.5
Street	75	4.6
Hotels	66	4.0
Other venues	26	1.6
Total	1642	100

### Sexually behaviors and drug use conditions

The median age of sexual debut was 18 years, and 21 years for the median age at which participants began sex work. Participants reported an average of one client a day, at an average charge of 100 RMB (16USD at the time of survey) per sex act. 213(13.0%) reported that they did not use a condom with the last male client. 810(49.3%) reported having a regular sexual partners (defined as either a husband or boyfriend of non-commercial partners) at the time of interview, with whom the majority (50.6%) of respondents reported that they never used condoms.

Out of the total sample of 1642 women, 212(12.9%) participants self-reported having ever used drugs, 233(14.2%) were tested positive in urine opiates, and 261(15.9%) were either self-reported having ever used drugs or be tested positive in urine opiates. Among those participants who self-reported having ever used drugs 184(86.8%) tested positive for urine opiates, for a Kappa statistic of 0.80. Of the 122 respondents who reported injection drug use in the previous 3 months, 75(61.5%) injected heroin only, while the remainder injected a mixture of heroin and other sedatives such as diazepam or promethazine. 16.4%(22/122) of those who reported recent injection drug use had shared syringes during that time.

### Incidence of HIV by BED-CEIA testing strategy

Total 10.2% (168/1642, 95%CI, 8.8–11.8) of FSWs were tested positive for HIV antibodies. All 168 positive specimens were tested by BED-CEIA, of which 16.7% (28/168) were identified as new HIV infections. The estimated crude incidence of HIV was 4.4(95%CI 2.8–6.0)/100PY, the McDougal-adjusted HIV incidence was 2.9(1.9–4.0)/100PY, and the Hargrove-adjusted HIV incidence was 3.2(95%CI 2.0–4.3) /100PY.

### Comparison of FSWs with and without Reported Drug Use History

As compared to non-illicit DU-FSWs, DU-FSWs were more likely to have worked in commercial sex for a longer duration (median 5.9 years. vs. median1.7 years, P<0.001), to have had more male clients in the previous week (median4 clients vs. median 2 clients, P<0.001), and to test positive for HIV antibody, *chlamydia trachomatis*(*CT*) and HSV-2(each P<0.001). These two groups did not differ in terms of other sexual behaviors. Results are shown in Table 2.

**Table 2:** Risk behaviors and HIV/STIs prevalence among FSWs by illicit drug-using condition.

Variables	Drug-using FSWs^#^ (n = 261, %)	Non drug-using FSWs (n = 1381, %)
Duration of commercial sex work: Median years(QIR)	5.9(2.3–9.4)	1.7(0.7–3.2)*
Number of male clients in the last week: Median number (QIR)	4(2–7)	2(1–5)*
Inconsistent condom use with clients in the last week	15.3	13.3
Consistent condom use with regular sex partners	17.0	19.4
Vaginal douching behaviors	77.3	78.7
Prevalence of HIV/STIs		
HIV	39.1	4.8*
Syphilis	11.9	7.9
Trichomonas vaginalis	9.6	7.9
Neisseria gonorrhoeae	6.1	5.5
Chlamydia trachomatis	20.3	10.1*
HSV-2	86.6	66.8*

* : p<0.001. #, Drug using FSWs included both of injection drug using FSWs(IDU-FSWs) and non-IDU-FSWs.

### Comparison of HIV risk factors for new versus established HIV infection

Univariate logistic regression identified current older age, reported history of drug use, longer duration in commercial sex work (greater than 2 years vs. 2 years or less), and HSV-2 infection as statistically significant risk factors for both new and established HIV infection (p<0.05 each). For recent infection as identified by BED-CEIA, however, lack of any formal schooling education, older age of sexual debut, and reported vaginal bleeding between menstrual periods were statistically significant risk factors (each p<0.05). While risk factors for established HIV infection were included being local permanent residence (vs. migrant FSWs), infection with *CT.* or *trichomonas vaginalis*, and reported lower abdomen pain in the past 12 months (each p<0.05). Results are shown in Table 3.

**Table 3:** Logistic regression analysis of HIV recent infection and long term infection related factors among Honghe FSWs.

Factors	Recent HIV infections (n = 28) vs. all other FSWs (n = 1474)				Established HIV infections (n = 140) vs. all other FSWs (n = 1474)			
	OR(95%CI)	*P*	aOR(95%CI)^†^	*P*	OR(95%CI)	*P*	aOR(95%CI)^‡^	*P*
Study site in Gejiu city (vs. in Kaiyuan city)	1.0(0.3–2.9)	0.974	-	-	0.6(0.3–1.0)	0.060	-	-
Residence places								
Local residence (vs. from other cities in Yunnan province)	1.3(0.5–3.3)	0.654	-	-	2.5(2.0–5.0)	<0.001	-	-
Local residence (vs. from outside of Yunnan province)	2.0(0.9–5.0)	0.094	-	-	10.0(3.3–25.0)	<0.001	-	-
Current age(yr.)								
21–25 (vs. 16–20)	1.5(0.5–4.7)	0.505	-	-	4.8(2.3–10.0)	<0.001	-	-
≥26 (vs. 16–20)	2.8(1.0–7.7)	0.047	-	-	8.6(4.3–17.3)	<0.001	-	-
No formal schooling vs. (received schooling)	3.3(1.4–7.8)	0.005	3.9(1.6–9.6)	0.003	0.9(0.5–1.7)	0.799	-	-
Illicit drug use* *vs*. (non-drug using history)	4.5(2.1–10.1)	<0.001	5.1(2.2–11.9)	<0.001	16.2(11.0–23.9)	<0.001	5.2(2.2–12.3)	<0.001
Age of first sex ≥18 years (vs. <18 years)	2.4(1.0–5.6)	0.042	-	-	1.3(0.9–1.8)	0.168	-	-
Duration in commercial sex work ≥2 years (vs. <2 year)	2.1(1.0–4.4)	0.049	-	-	5.5(3.8–8.2)	<0.001	4.0(2.6–6.0)	0.001
Failed use condom use with last clients (vs. used condom)	1.4(0.5–3.8)	0.474	-	-	1.5(1.0–2.4)	0.074	-	-
STDs infection status								
HSV-2 infection (vs. without HSV-2 infection)	3.3(1.1–9.5)	0.020	-	-	3.9(2.3–6.6)	<0.001	3.3(2.0–5.7) (1.2-3.8)	<0.001
Syphilis infection (vs. without Syphilis infection)	1.4(0.4–4.7)	0.591	-	-	1.5(0.9–2.6)	0.145	-	-
*NG*# infection (vs. without *NG* infection)	1.4(0.4–4.0)	0.554	-	-	0.6(0.2–1.1)	0.071	-	-
CT^&^ infection (vs. without CT infection)	1.1(0.5–2.4)	0.899	-	-	2.1(1.3–3.4)	0.002	-	-
*Trichomonas vaginalis* infection (vs. without *Trichomonas) vaginalis )*infection	1.4(0.4–4.2)	0.511	-	-	1.6(1.0–2.6)	0.049	2.4(1.4–3.9)	0.001
Vaginal bleeding symptoms between menstrual period	4.4(1.6–12.0)	0.001	5.2(1.9–14.5)	0.001	1.4(0.7–2.9)	0.347	-	-
Lower abdomen pain in past 12 months	2.1(1.0–4.4)	0.060	-	-	1.6(1.1–2.3)	0.017	-	-

#, NG: *Neisseria gonorrhoeae*; &, CT: *Chlamydia trachomati*; *, self-reported once used illicit drugs or be tested urine opiates positive;†, adjusted for residence places(local residence, from other cities in Yunnan province, from outside of Yunnan province), current age(16–20 years, 21–25 years, ≥26 years); ‡, adjusted for study sites(Gejiu city, Kaiyuan city), residence places(local residence, from other cities in Yunnan province, from outside of Yunnan province) and current age(16–20 years, 21–25 years, ≥26 years).

Multivariate logistic regression was carried out by controlling for all of the social demographic characteristics found to be statistically significantly at an alpha level of 0.20 (for a two tailed test) associated with HIV infection in the univariate analysis (Table 3). The analysis found that without having formal schooling (aOR, 3.9, 95%CI, 1.6–9.6), reported vaginal bleeding between menstrual period in past 12 months (aOR, 5.2, 95%CI, 1.9–14.5), and a reported history of drug use (aOR, 5.1, 95%CI, 2.2–11.9) were all significantly associated with recent HIV infection (each p<0.05) (table 3). Variables that were significantly associated with established HIV infection included reported history of drug use (aOR, 5.2, 95%CI, 2.2–12.3), longer duration of sex work (≥2 years vs. less than 2 years; aOR, 4.0, 95%CI, 2.6–6.0), HSV-2 infection (aOR, 3.3, 95%CI, 2.0–5.7), and *Trichomonas vaginalis* infection(aOR, 2.4, 95%CI, 1.4–3.9) (each p<0.05) (table 3).

## Discussion

Our results show that a history of drug use poses significant risks for both new and established HIV infection among FSWs, underscoring the importance of integrated prevention services to address dual risk behaviors of drug using FSW. Crude HIV-incidence among Honghe FSWs was 4.4 per 100PY, far higher that rates reported in FSW populations in neighboring Sichuan province (1.0/100 PY) [31], but similar with that of Thailand FSWs (4.3/100 PY) [32], and relatively lower compared to that rates of FSWs fromZaire (8/100 PY) [33], and the US(12-18/100 PY) [34]. It is also worth noting that prevalence rates from this same population have been rising over time, from 8.3% in 2006 to10.3% in 2007 and 11.3% in 2008, though some of these differences may be due to changes in sampling strategies or other external factors [12], [13], [20].

Our results also highlight a complex but undeniable relationship between drug use and higher risk sexual practices among FSW (Table 2). That DU-FSW reported larger numbers of clients in the past week may be due to the greater demand for money generated by their drug use, or possible evidence of drugs being exchanged for sex in situations often described as “survival sex” (though this specific question was not asked in our survey) [35]. Aside from the risk of HIV infection posed by drug use alone, DU-FSW may be at greater risk for sexual HIV acquisition through exposure to greater numbers of partners or more frequent sex, and the compromised ability to negotiate safe sex while under the influence of drugs [17], [18], [20], [21]. Although our findings confirm the conclusions of a recent literature review which reported that DU-FSW are at far greater risk of HIV infection [10], our analysis also raises many questions about the nature of the relationship between drug use and risky sex among sex workers. Research with the same FSW population is ongoing in order to better understand the potential role that sex work among women and illicit drug use play in the initiation of the other [36].

Risk factors analysis for recent HIV infection as distinguished by BED-CEIA testing strategies among FSWs can provide insight into evolving risk behavior patterns in the population and further guide HIV control measures. That a reported history of drug use was an important common risk factor for both recent (aOR = 5.1, 95%CI, 2.2–11.9) and established HIV infection (aOR = 5.2, 95%CI,2.2–12.3) underscore the salience of drug use for HIV infection; however, the fact that STI's and symptoms were also common risk factors for both new and established HIV infection shows that other STI may have a persistent moderating effect on HIV acquisition that is independent from that of drug use. This highlights the importance of combination prevention to simultaneously address the HIV acquisition risk posed by untreated STI as well as drug use.

A comparison of variables identified as significant predictors of recent HIV infection versus established HIV infection in the multivariate analysis provides several new insights into patterns of HIV acquisition among the FSW in our study. First, longer duration of sex work was associated with established infection but not with recent HIV infections. The fact that recent infection was not associated with longer duration of sex work may reflect the fact that HIV acquisition requires a minimum of cumulative exposures, in the course of which the likelihood of acquiring other STIs also increases the risk of HIV infection.

Additionally, the lack of formal schooling was predictive of recent, but not established, HIV infection among the FSW surveyed. The fact that education is a salient predictor only of recent infection may be the result of the more educated subgroups of FSW becoming better able to protect themselves from HIV over time. Such a phenomenon might occur if FSW directed HIV education campaigns had a differential impact on educated versus uneducated FSW. This could occur if, for instance, education campaigns failed to reach the less educated subgroups, if the campaigns failed to empower less educated FSW with culturally relevant prevention methods, or if less educated FSW are less economically and socially capable of protecting themselves from HIV in spite of successful prevention efforts. Prevention efforts that tailor to the highly diverse subgroups of FSW [31], [37] and rigorously assess the efficacy of these interventions for the less educated – and therefore more vulnerable – FSW is imperative for effective HIV control efforts in this community.

Our study has several limitations. As with all cross-sectional studies, we are unable to infer causal relationship between identified risk factors and HIV infection. Secondly, since we do not have any data on the HIV prevalence among the client pool of the FSW population from which we sampled, we also have no way to explore the validity of the common hypothesis that FSW are a “source” of HIV infection into the general population through their male client partners. Thirdly, most of FSWs participants were recruited from KTV venues which were usually had lower risk behaviors, so the HIV epidemic may be underestimated. Fourthly, the small number of recent infections (n = 28) may have compromised the ability of the model to accurately predict the probability of such events [38], and may case insufficient statistical power to test effect measure modification hypotheses, so the results need to be interpreted with caution. Finally, reporting bias is common in research of socially sensitive issues such as sex work or drug use. Although strategies such as confidential and anonymous interviews with trained staff were carried out, under reporting of high risk behaviors is likely.

In summary, this study of the HIV epidemic among female sex workers (FSWs) in high prevalence areas where injection drug use is also widely documented, identified relevant drivers of incident HIV. Our analysis also allowed us to evaluate and compare factors correlated with recent versus established HIV infection, which is important for understanding dynamic drivers of the epidemic. A more time relevant and nuanced understanding of the epidemic in such a way as to better inform novel prevention methods to curb the persistent spread of HIV among higher risk groups in Yunnan province. Combining sexual risk reduction, condom promotion and improved STI treatment strategy is urgently needed to reduce HIV acquisition among FSWs and the spread of HIV to the general population in southern China.
